# Eating Disorders during the pandemic COVID-2019

**DOI:** 10.1192/j.eurpsy.2022.1497

**Published:** 2022-09-01

**Authors:** B. Rodríguez Rodríguez, I. Santos Carrasco, M. Fernández Lozano, M.J. Mateos Sexmero, N. Navarro Barriga, C. De Andrés Lobo, T. Jiménez Aparicio, C. Vallecillo Adame, G. Guerra Valera, A. Gonzaga Ramírez, M. Queipo De Llano De La Viuda, J. Gonçalves Cerejeira, C. Valdivieso Burón

**Affiliations:** 1 Hospital Clínico Universitario, Psiquiatría, Valladolid, Spain; 2 Hospital Clínico Universitario, Psiquiatría, VALLADOLID, Spain

**Keywords:** Pandemic COVID-19, Eating Disorders, lockdown

## Abstract

**Introduction:**

Concerns about health and fitness during lockdown may serve as a trigger for eating disorders in vulnerable individuals. Other risk factors may also include increased use of social networks and comparison with beauty ideals. Isolation, loneliness and problems with emotional regulation may lead people to reduce food intake by giving them a greater sense of control.

**Objectives:**

Emphasise the relevance of the increase in the incidence of Eating Disorders (ED) cases during the pandemic.

**Methods:**

Review of the scientific literature based on a relevant clinical case.

**Results:**

14-year-old female, residing with her mother. She reports that from the beginning of COVID-19 confinement she became obsessed with leading a healthier life, starting to restrict food, limiting fats and carbohydrates, and having also started compulsive physical activity (approximately 4 hours of aerobic exercise per day), without associated purging behaviours. She also acknowledges eating small amounts (although she minimises this aspect) and controlling all calories, stating that food and practices aimed at “staying healthy” now dominate her life. Her previous BMI was 18, with a current BMI of 11.7.

**Conclusions:**

Patients suffering from ED, who often have poor knowledge of their illness and find social-emotional communication difficult, may delay seeking help. Studies suggest the relevance of identifying specific vulnerability factors among ED patients in confinement in order to develop preventive strategies and personalised treatment approaches.

**Disclosure:**

No significant relationships.

